# Oxidative stress in the brain is regulated by social status in a highly social cichlid fish

**DOI:** 10.3389/fnbeh.2024.1477984

**Published:** 2024-11-26

**Authors:** Peter D. Dijkstra, Robert J. Fialkowski, Brady Bush, Ryan Y. Wong, Travis I. Moore, Ashley R. Harvey

**Affiliations:** ^1^Department of Biology, Central Michigan University, Mount Pleasant, MI, United States; ^2^Neuroscience Program, Central Michigan University, Mount Pleasant, MI, United States; ^3^Institute for Great Lakes Research, Central Michigan University, Mount Pleasant, MI, United States; ^4^Department of Biology, University of Nebraska at Omaha, Omaha, NE, United States

**Keywords:** oxidative stress, cichlid, social stress, reproduction, dominance hierarchies, antioxidant capacity, territoriality

## Abstract

Social stress can increase reactive oxygen species and derail antioxidant function in the brain, which may contribute to the onset and progression of mental health disorders. In hierarchical species, repeated social defeat can raise oxidative stress in the brain. However, how oxidative balance in the brain is regulated across different levels in a social hierarchy is unknown. Here, we study the effect of social status on patterns of oxidative stress across several brain divisions in a highly social cichlid fish, *Astatotilapia burtoni*. In this species, dominant males are territorial, brightly colored, and reproductively active while subordinate males are not. We measured several markers of oxidative stress in macrodissected brain divisions in dominant and subordinate males. We found that dominant individuals had lower oxidative DNA damage (8-OhdG) in the midbrain while also having increased total antioxidant capacity in the midbrain and hypothalamus. However, in dominant males, oxidative DNA damage tended to be higher in the hypothalamus while total glutathione levels were lower in the telencephalon compared to subordinate males. Finally, we found that indicators of reproductive activity (gonadosomatic index and social behavior) were co-regulated with antioxidant function or oxidative damage in the telencephalon. Combined, our results suggest that social status and activation of the reproductive system regulate oxidative balance in the brain in a highly brain division specific manner.

## Introduction

Oxidative stress occurs when reactive oxygen species (ROS) overwhelm the antioxidant and repair systems, resulting in oxidative damage and cellular dysfunction (Janicki-Deverts et al., 2009; Beaulieu et al., 2014; Georgiev et al., 2015; Border et al., 2019). The brain is particularly vulnerable to oxidative stress due to its high oxygen consumption, lipid-rich environment, and relatively low antioxidant defense mechanisms (Kandlur et al., 2020). Accordingly, cumulative oxidative damage in the brain has been implicated in the onset and progression of depression and neurodegenerative disease (Black et al., 2015; Blesa et al., 2015; Cobb and Cole, 2015; Do et al., 2015).

It is well recognized that environmental stressors can increase ROS and derail antioxidant function (Ghezzi et al., 2018; Münzel et al., 2018; Rao et al., 2018). In modern human societies, environmental stressors are mostly social in nature, such as conflict at home, social marginalization, or social isolation (Almeida, 2005; Koenen et al., 2010; Park et al., 2015; Neblett, 2019). A certain degree of social stress is physiologically tolerated but when social stress is persistent (such as prolonged social marginalization or daily social conflict) it can contribute to the development of disease (Picard and McEwen, 2018). Therefore, there is a critical need to understand how social stress influences oxidative stress in the brain and how social stress-induced oxidative insults are regulated by innate defense mechanisms (Walton et al., 2012; Schiavone et al., 2013).

In social animals, competition for access to mates and resources may lead to the emergence of dominance hierarchies (Tibbetts et al., 2022). Individuals with high social status are more aggressive and monopolize mates and resources while those with low social status behave submissively and have fewer reproductive opportunities (Snyder-Mackler et al., 2020). Past studies have shown that social stress arising from social defeat (which leads to low social status) leads to increases in oxidative stress in specific brain regions that are relevant to mood disorders (Schiavone et al., 2013; Salim, 2014; Fedoce et al., 2018). For example, repeated social defeat stress resulted in increased oxidative stress in the hippocampus but not the amygdala and cortex in rats (Patki et al., 2013). However, past studies considered a small subset of brain regions relevant to mood disorders (Patki et al., 2013), used only one marker of oxidative stress (Ibi et al., 2017; Lehmann et al., 2019), and only examined individuals with low social status using brief dyadic encounters in resident-intruder tests (Bouvier et al., 2017; Lim et al., 2020). However, hierarchy establishment in an experimental setting featuring a steady-state group leads to a more realistic and ethologically relevant social structure (Larrieu and Sandi, 2018). Finally, while low social status is clearly stressful, in these group settings high social status can also be challenging since maintaining high social status is psychologically and metabolically demanding due to intense competition for rank and increased investment in reproduction (Archie et al., 2012; Creel et al., 2013; Anderson et al., 2021). However, how social stress alters redox state across the brain of both low and high ranked individuals in stable social hierarchies is unknown (Gonçalves et al., 2008).

Social status is often linked to distinct behavioral and neuroendocrine profiles. Low-ranked individuals typically exhibit increased activation of the hypothalamic–pituitary adrenal (HPA) axis as indicated by high levels of circulating glucocorticoids (Abbott et al., 2003; Goymann and Wingfield, 2004). By contrast, in highly ranked individuals the hypothalamic–pituitary-gonadal (HPG) axis, which controls androgen release and activates the reproductive system, is upregulated (Goymann, 2009; Maruska and Fernald, 2011). Most attention has been given to social stress-induced hyperactivity of the HPA axis and glucocorticoid-mediated effects of oxidative stress in the brain (Patki et al., 2013; Ibi et al., 2017). However, the HPG axis also responds to social stress, with androgen levels increasing when individuals are challenged by others in the hierarchy (Zilioli and Watson, 2014). Less attention has been given to the role of the HPG axis in modulating brain oxidative stress, despite reproduction and androgens having direct effects on oxidative balance in a range of tissue types, including the brain (Bueno et al., 2017; Snyder et al., 2018; Chunchai et al., 2019; Duong et al., 2020; Muthu and Seppan, 2020). Given that rank regulates activation of the HPG axis in many social species, dominance hierarchies are ideally suited to explore the link between HPG axis activity and brain oxidative stress within a social context.

**Figure 1:**
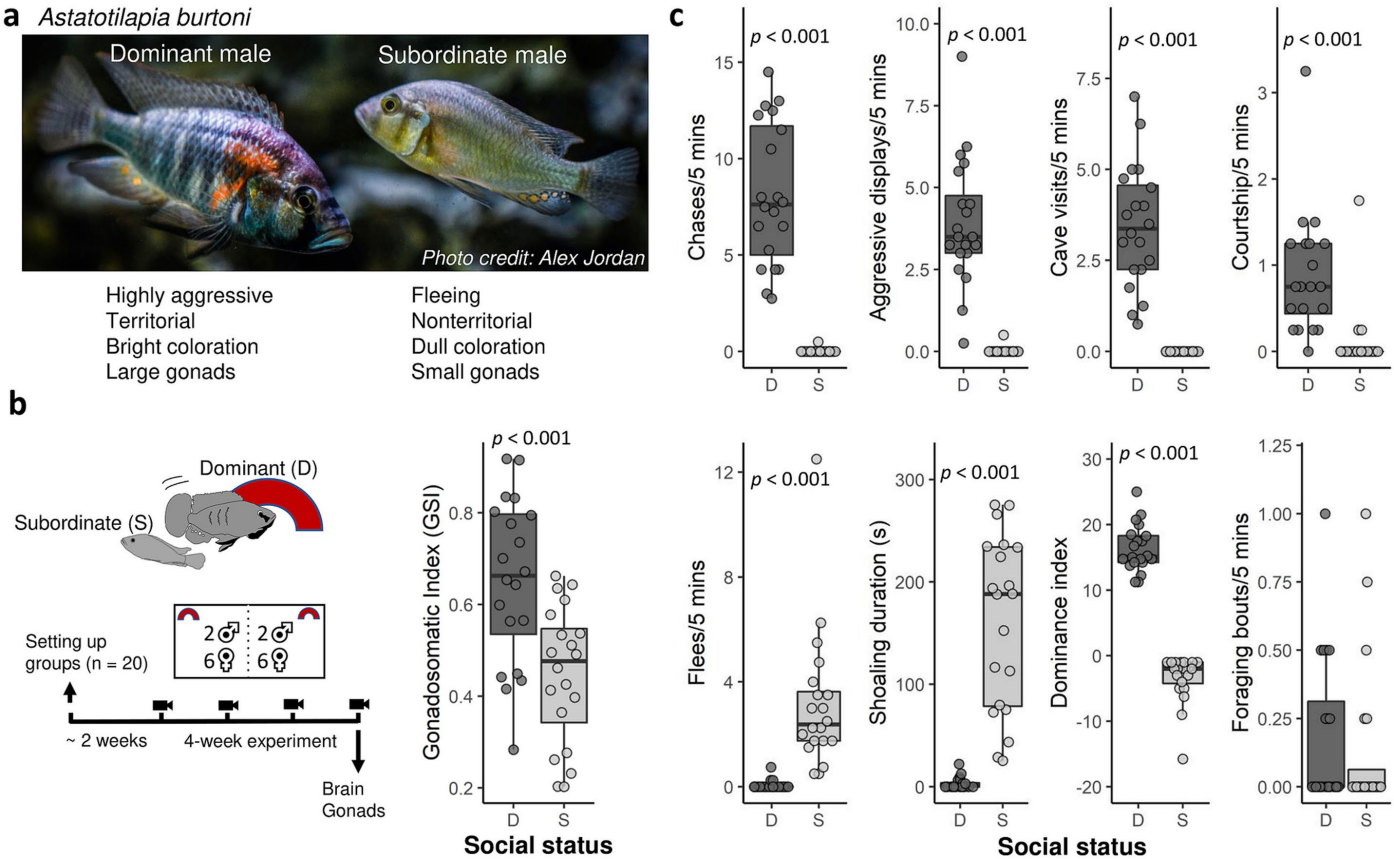
**(a)** Picture of a dominant and subordinate *Astatotilapia burtoni* male. **(b)** Experimental timeline and gonadosomatic index in dominant and subordinate males. **(c)** Behavior in dominant (D, *n* = 20) and subordinate (S, *n* = 20) males. Shown are the number of behaviors per 5 min (chases, aggressive displays, cave visits or flowerpot entries, courtship), time spent shoaling amount during the 5-min observation period, and the dominance index (aggressive behavior and courtship minus flees) averaged across the four weekly filming sessions prior to tissue sampling. Bold lines indicate medians. Boxes enclose 25th to 75th percentiles. Error bars enclose data range, excluding outliers.

The highly social cichlid fish *Astatotilapia burtoni*, forms social hierarchies in the laboratory mimicking those found in the wild (Maruska and Fernald, 2018). Male *A. burtoni* exist as two reversible phenotypes (Figure 1a) dominant and subordinate males (O’Connell and Hofmann, 2012; Maguire et al., 2021). Dominant males have a highly active HPG axis (as indicated by high androgen levels and large gonads), are brightly colored, aggressive, and establish territories where they court females (Border et al., 2021). Subordinate males have a downregulated HPG axis, are non-territorial, and drab in color. Previous studies have indicated that in *A. burtoni*, dominant males have higher circulating levels of reactive oxygen metabolites, a marker of oxidative damage, than subordinate males (Border et al., 2019, 2021; Fialkowski et al., 2021, 2022), and a similar pattern was found in two other haplochromine cichlid species (Dijkstra et al., 2011). Our model species *A. burtoni* has unique properties that make it ideal to study the effect of the social environment and competition on brain oxidative stress: (a) the social dominance hierarchy is highly tractable since there are clear phenotypic signatures of social status (Fernald and Hirata, 1979; Alward et al., 2020), and (b) social status mostly results from direct competitive interactions (Dijkstra et al., 2022) and is not significantly complicated by other factors such as affiliative interactions, coalitionary behavior, or hereditary determinants of social status (Snyder-Mackler et al., 2020).

In the current study, we assigned social status and quantified social dominance behavior in experimental mixed-sex groups of *A. burtoni*. We then analyzed markers of oxidative stress (oxidative damage and antioxidant function) in macrodissected brain divisions in dominant and subordinate males. We hypothesize that oxidative stress varies by social status as well as by indicators of HPG axis activity, such as relative gonad size and social dominance behavior.

## Materials and methods

### Animals

All animals used in this experiment were adult *A. burtoni* (approx. 15–18 months; standard length 57–81 mm; weight 5.5–15.5 g; total males *n* = 40) from a laboratory-bred population that was derived from wild-caught fish collected from the Burundi coast of Lake Tanganyika (Fernald and Hirata, 1977). Fish were housed in aquaria containing gravel substrate and kept at 28°C with a 12-h light/dark cycle containing a 10-min dusk and dawn period to mimic natural settings. Fish were fed cichlid flakes (Omega Sea LLC, Painesville, Ohio) each morning between 8:00 and 10:00 am. All males were individually tagged with colored beads through the dorsal musculature using a stainless-steel tagging gun. All animal care procedures were approved by Central Michigan University Institutional Animal Care and Use Committee (IACUC protocol 15–22) and were in compliance with the US National Research Council’s Guide for the Care and Use of Laboratory Animals, the US Public Health Service’s Policy on Humane Care and Use of Laboratory Animals, and Guide for the Care and Use of Laboratory Animals.

### Experimental design and tissue collection

A visual of the experimental timeline is shown in Figure 1b. We divided experimental 110-liter tanks in half widthwise with clear, perforated acrylic barriers into two compartments as described previously (Fialkowski et al., 2021). To assign social status based on size difference, we placed one larger male and one smaller male (weight difference: 2.385 ± 0.930 g; standard length difference: 6.95 ± 1.76 mm) along with 6 females in each experimental compartment. Assignment of status by size was successful as each larger male was dominant while the smaller male was subordinate throughout the duration of the experiment (the hierarchy was typically established in 1 day, with the dominant males defending a flowerpot, expressing a dark eye bar while subordinate males were more cryptically colored and shoaled with females). We set up a total of 30 groups (each containing 2 males and 6 females). Of these 30 groups, 8 groups were lost during the experiment either due to a male that died (in 6 groups the smaller male died) or because the group was adjacent to a group where a male died (2 cases). We further excluded two groups where there was no clear hierarchy with both males assuming dominance status. Our final sample size was 20 groups where the largest male became dominant (*n* = 20) and the smaller subordinate (*n* = 20). Males were weighed and their standard length was measured prior to being added to experimental compartments. The initial body mass of dominant males was 10.75 ± 2.098 g (range: 7.7–15.5 g) with a standard length (SL) of 71.75 ± 4.84 mm (range: 63–81 mm). The initial body mass of subordinate males was 8.705 ± 1.868 g (range: 5.5–11.7 g) with a standard length of 65.5 ± 4.68 mm (range: 57–74 mm). In each compartment, one halved terracotta flowerpot was added to promote territoriality in the dominant male. Fish were able to interact physically with members in their own group and visually with those in the adjacent compartment. This allowed for the full expression of social behaviors including aggressive interactions between dominant males between compartments. Fish were allowed to settle in their experimental tank for 2 weeks. We then started weekly filming of each group for behavioral quantification (5 min) between 8 and 10 a.m. for 4–5 weeks. The final recording was made on the morning of tissue collection. During the 4–5 weeks experiment, we recorded the social status of all experimental fish three times per week. Social status was determined by characterizing males as dominant or subordinate, and all males retained the same social status throughout the experiment.

From each experimental group (*n* = 20), we collected tissue and blood from the dominant male and subordinate male at the end of the experiment (i.e., after the final recording in week 4 or 5). Fish were weighed and measured for standard length before blood and tissue collection. Blood was drawn through the caudal vein using heparinized 26-gauge butterfly needles (Terumo) and plasma was saved for another project. Immediately after blood draw, males were euthanized by spinal cord dislocation. Brains were harvested and rapidly macrodissected using a razor blade into four brain divisions: telencephalon (telencephalon and olfactory bulbs), hypothalamus, midbrain (tectum and thalamus), and hindbrain (cerebellum and hindbrain). When separating the telencephalon from the rest of the brain we made a transverse cut at the anterior commissure. The telencephalon cut likely contained a significant part of the parvocellular portion of the preoptic area. The hypothalamic cut included the magno−/gigantocellular portion of the preoptic area, and it likely also contained a small part of the parvocellular portion. Each brain division was flash frozen in liquid nitrogen then immediately placed in a 1.5 mL tube on a Coolrack block (Biocision, Larkspur, CA, USA) on dry ice. Gonads were weighed for gonadosomatic index data. The experiment was carried out between October 2020 through December 2020. All experimental procedures were done blind with respect to social status.

### Behavioral and morphological analysis

Using the videos from the previous 4 weeks before tissue collection we quantified frequencies and/or duration of behavior using Behavioral Observation Research Interactive Software [BORIS] (Friard and Gamba, 2016) as described previously (Fialkowski et al., 2022). As indicators of social dominance behaviors, we quantified the number of chases, aggressive display behaviors (border threat and lateral display), cave visits, and courtship displays. We also scored the frequency of fleeing and duration of shoaling (both are indicators of subordinate behavior) and the frequency of foraging behavior. Behavioral recording was done by a single observer.

### Choice of markers of oxidative stress

In each brain division sample, we measured two markers of antioxidant capacity and one marker of oxidative damage. Total antioxidant capacity (TAC) is a measure of both enzymatic and non-enzymatic antioxidant capacity. We also measured glutathione, an important antioxidant (detailed below). In each brain division sample, we evaluated oxidative DNA damage by measuring levels of 8-hydroxy-2′ -deoxyguanosine (8-OhDG). Below is a description of all the assays. All samples were run in duplicate, and we included a pooled tissue sample to evaluate interplate variability for all assays. For all assays, the intra-assay CV (coefficient of variation) and the interassay CV were typically below 5 and 13%, respectively unless indicated otherwise. For all assays we used clear flat-bottom 96-well plates unless indicated otherwise.

### Tissue preparation

We homogenized each brain division sample and prepared different aliquots for total antioxidant capacity (TAC), glutathione, and oxidative DNA damage assay using a generalized homogenization buffer. Each brain division sample was homogenized in tissue homogenization buffer using a motorized pestle mixer (VWR) in a 1.5 mL centrifuge tube. The homogenization buffer consisted of potassium phosphate buffer (50 mM PBS (pH 7.4) and 0.5 mM EDTA) with a Protease Inhibitor Cocktail (Sigma-Aldrich P2714) added at a 1:10 dilution. We used ~10 μL buffer per 1 mg tissue. Once homogenized, the tissue homogenates were aliquoted into 3 separate 0.5 mL centrifuge tubes for further samples preparations for TAC, glutathione, and DNA damage (volumes of tissue homogenates for each assay are indicated below).

For TAC, 40 μL of tissue homogenate was mixed with 40 μL lysis buffer (20 mM Tris–HCl, 137 mM NaCl, 1% NP-40, 10% glycerol, 2 mM EDTA). This mixture was then centrifuged at 4°C at 10,000 g for 10 min as described elsewhere (Fialkowski et al., 2021). The supernatant was removed and stored at −80°C in separate tubes for TAC and protein quantification.

For glutathione, 60 μL tissue homogenate were centrifuged at 10,000 g for 10 min. Following centrifugation, 45 μL supernatant was transferred to a new tube and mixed with 45 μL 5% 5-sulfosalicylic acid dihydrate (SSA) solution and incubated at 4°C for 10 min. Samples were then centrifuged at 10,000 g for another 10 min at 4°C. The supernatant was removed and stored at −80°C in separate tubes for glutathione and protein quantification.

For oxidative DNA damage (8-OHdG), 150 μL of tissue homogenate was centrifuged at 4°C at 10,000 g for 10 min. The supernatant was then discarded, and the remaining pelleted tissue homogenate was stored at −80°C for quantifying DNA damage.

### Protein quantification

Protein concentration of each prepared brain division sample for glutathione and TAC was measured with a bicinchoninic acid (BCA) Protein Assay kit (Pierce, Rockford IL) following the manufacturer’s protocol. Frozen supernatant was thawed on ice and diluted 1:4 with buffer used in their respective sample preparation. We used 10 μL of this diluted supernatant in the assay. Absorbance was read by a plate reader (Epoch2T, Biotech Instruments, Winooski, VT, USA).

### Total antioxidant capacity

Total antioxidant capacity (TAC) was measured via an Oxygen Radical Absorbance Capacity (ORAC) assay as previously described (Border et al., 2019; Fialkowski et al., 2021). For each sample, the brain division supernatant was standardized to 150 ug protein/mL using the 50:50 lysis-homogenization buffer. We took 50 μL of this 150 ug protein/mL sample and diluted it again 1:2 using PBS to a total volume of 100 μL. We used 20 μL of diluted sample for the ORAC assay. For each brain division sample, ORAC was reported as μmol TE/μg protein. Absorbance was read by a plate reader (Spectramax M3, Molecular Devices, Sunnyvale, CA, USA).

### Glutathione

We measured glutathione (GSH) in homogenized brain division samples using a glutathione fluorescence kit (Arbor Assays, Ann Arbor, MI, USA) per manufacturer’s protocol. Samples were first diluted 1:2.5 with kit assay buffer to reduce Aqueous 5-sulfo-salicylic acid dihydrate (SSA) concentration to 1%. Using 50 μL of diluted sample, samples and standards were then added to the plate (Corning 3,686 black half-area microtitre plate), mixed with 25 μL ThioStar detection reagent, and incubated at room temperature for 15 min. Endpoint fluorescence was read using a plate reader (Spectramax M3, Molecular Devices, Sunnyvale, CA, USA) to obtain reduced GSH. We then mixed each sample with 25 μL kit reaction mixture and incubated it for 15 min at room temperature to convert all oxidized GSH dimers (GSSG) in sample to reduced GSH. Following incubation, samples were again measured via endpoint fluorescence reading using the same plate reader mentioned above to obtain total GSH. Due to technical limitations, we were only able to measure total GSH (which is reduced GSH as well as oxidized GSH measured as reduced). All reported values were standardized to the protein concentration in each sample (total GSH/ μg protein).

### Oxidative DNA damage

For each brain division sample, we measured oxidative DNA damage (8-OHdG) following a previously described protocol (Fialkowski et al., 2021) which we modified to allow for measurements of small tissue samples. DNA was extracted from homogenized brain tissue samples using a DNA extraction kit (Zymo quick-DNA miniprep plus kit, Irvine, CA, USA) per manufacturer’s protocol. Purified DNA samples were extracted by adding 200 μL of DNA elution buffer and incubating at room temperature for 5 min before centrifuging at 17,000 g for 1 min to obtain optimal DNA concentrations. We then used ethanol precipitation to increase DNA concentration in each sample through addition of 19 μL 3 M Sodium acetate (pH 5.2) and 475 μL cold ethanol. Samples were then stored at −20°C overnight before undergoing 30-min centrifugation at top speed at 4°C. All ethanol was then removed, and the DNA was reconstituted in 15 μL elution buffer. After ethanol precipitation, the total DNA concentrations were determined using a BioTek take3 reader in an Epoch2T microplate reader using the Nucleic Acid Quantification software provided. We standardized all DNA samples to 200 ng/μL and stored sample at 4°C until digestion.

DNA samples were digested following a modified protocol from Quinlivan and Gregory (2008) and as described previously (Fialkowski et al., 2021). Following digestion, samples were stored at −20°C until use in DNA damage assay.

DNA damage (8-OHdG) was measured with a DNA damage ELISA kit (StressMarq, Biosciences Inc., Victoria, BC, Canada) following manufacturer’s protocol. We diluted each digested sample in sample and standard diluent to ensure samples were put on the plate at a standardized concentration (midbrain and hindbrain: plate concentration 8.33 ng/μL; telencephalon and hypothalamus: plate concentration 7.14 ng/ μL) using the DNA concentrations measured after ethanol precipitation. Results are reported as ng damage (8-OHdG) / ng DNA.

### Statistical analysis

As a measure of investment in the reproductive (HPG) axis, we calculated the gonadosomatic index as GSI = [gonad mass/body mass] * 100 (Bolger and Connolly, 1989). Upregulation of the reproductive axis is also associated with increased territorial behavior whereas downregulation of this axis is linked to fleeing behavior. To quantify this link between investment of the reproductive axis and behavior, we calculated a dominance index for each fish through subtracting the number of flees from the sum of the aggressive behaviors, courtship, and cave visits (Maruska et al., 2013). All analyses were conducted in RStudio using the R packages lme4, lmerTest, MASS (Bates et al., 2015), glmmTMB (Brooks et al., 2017), and emmeans. We identified and excluded outliers in our oxidative stress data based on Tukey’s rule (between 0 and 3 values were excluded per measurement with most measurements having no outliers).

Most behaviors were (almost) exclusively exhibited by either subordinate or dominant males. We therefore compared total aggression (sum of chases and aggressive display behaviors), cave visits, courtship, fleeing events, and shoaling duration, averaged for each male across the 4-week observation period, using Wilcoxon Signed-Rank (WSR) on paired samples (comparing dominant and subordinate males in the same group).

We used linear mixed models (LMMs) with a maximum-likelihood protocol with social status and/or TAC as fixed effects and pair as a random effect. We compared GSI between dominant and subordinate males using LMMs with social status as fixed effect. We ran similar models to compare levels of TAC, DNA damage, and total GSH between dominant and subordinate males for each brain division separately. To test for social status specific regulation of oxidative balance, we tested, for each brain division separately, whether variation in oxidative DNA damage was explained by TAC in a social status-specific manner using LMM with social status and TAC as interaction effect. In all analyses we also included body weight but this effect was only retained if significant. To evaluate the validity of our models, we examined the residuals, qqplots, and plots of predicted values versus residuals.

To examine whether HPG axis regulation is related to patterns of oxidative stress in the brain, we examined covariance patterns of the different measures of oxidative stress with two markers of HPG axis activity, the dominance index and gonadosomatic index (GSI). We carried out this analysis in dominant and subordinate males separately and created heatmaps using the R package gplots. In dominant males, we also included aggressive display (sum of lateral display and border threat) and chases in the analysis. The behavioral data used in the covariance analysis was based on the final video recorded immediately before tissue collection, closest to the physiological sampling period. We carried out hierarchical cluster analysis to determine clusters that were co-regulated. *p-*values for each cluster were obtained through multiscale bootstrap resampling (10,000 steps) using the pvclust package with the Ward clustering method (Suzuki and Shimodaira, 2006). A significance level (*α*) of 0.05 was used for all tests. We report mean ± SE for our model estimates.

## Results

### Behavior and gonadosomatic index

Males did not change social status during the entire duration of the experiment. We calculated the average rate or duration of different behaviors across the four weekly five-minute observations (Figure 1c). Compared to subordinate males, dominant males exhibited more chases (WSR test, *V* = 210, *p* < 0.0001), more aggressive display behaviors (*V* = 210, *p* < 0.0001), more cave visits (*V* = 210, *p* < 0.0001), and more courtship behavior than subordinate males (*V* = 190, *p* < 0.0001). Conversely, subordinate males performed more flee behavior (WSR test, *V* = 0, *p* = 0.0009) and showed a greater duration of shoaling behavior (*V* = 0, *p* = 0.0009) than dominant males. The dominant index (DI, sum of aggressive and courtship behavior minus flees) was significantly higher in dominant than subordinate males (WSR test, *V* = 210, *p* < 0.0001). There was no difference in foraging behavior between dominant and subordinate males (WSR test, *V* = 46.5, *p* = 0.57). These results confirm that dominant males direct territorial aggression towards subordinate males. Dominant males had greater GSI than subordinate males (LMM, −0.20474 ± 0.04457, t_20_ = −4.594, *p* = 0.000176, Figure 1b), confirming that the former had an activated reproductive system.

### Effect of social status on patterns of oxidative stress in the brain

**Figure 2:**
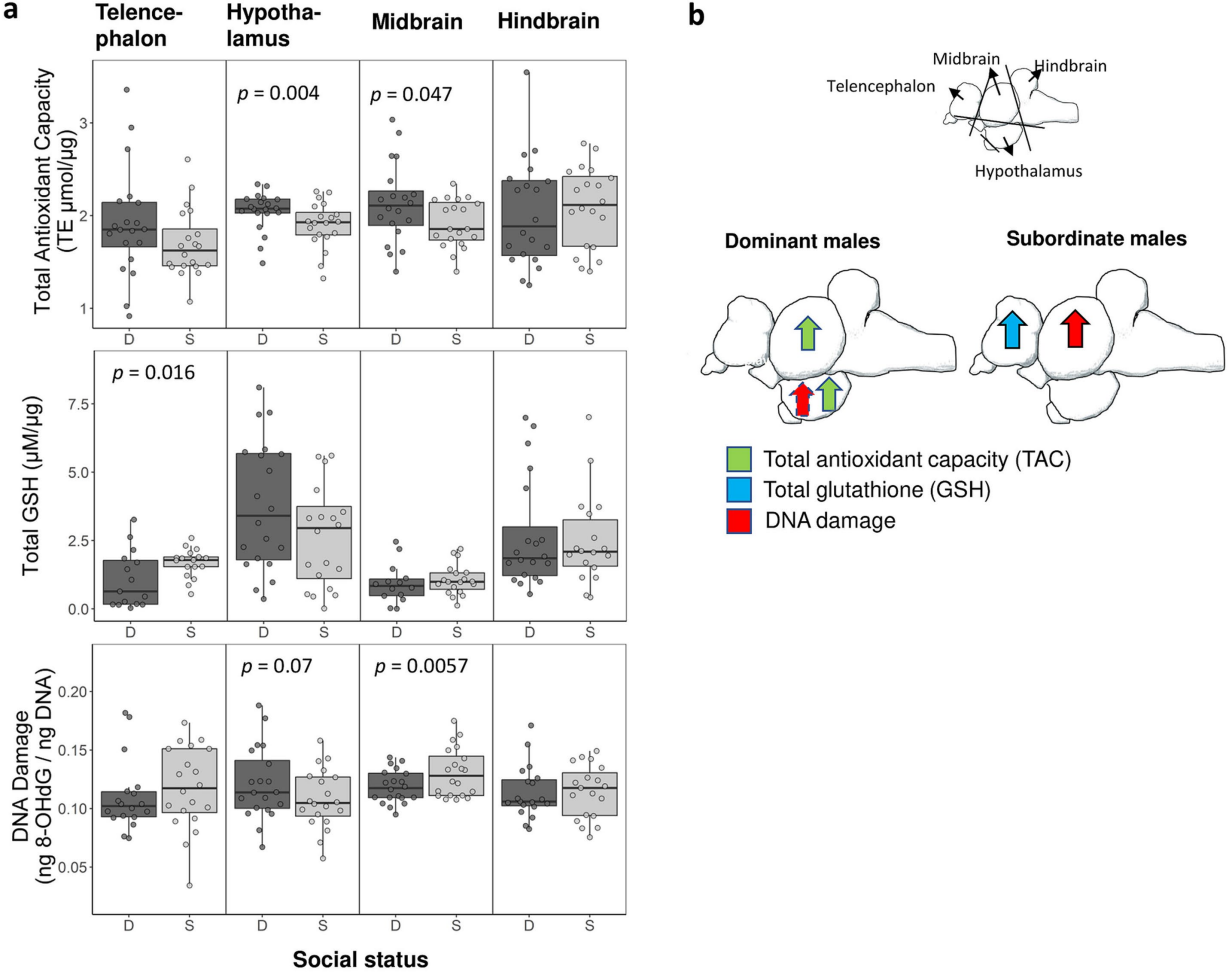
**(a)** Measurements of oxidative stress in dominant (D) and subordinate (S) males in each of the four brain divisions. Shown is data for total antioxidant capacity, total glutathione (GSH), and oxidative DNA damage. We collected samples from 20 dominant and 20 subordinate males but sample sizes ranged across assays (final sample sizes are shown in Table 1). Boxes enclose 25th to 75th percentiles. Error bars enclose data range, excluding outliers. Dots are data points. TE: Trolox equivalents. **(b)** Summary schematic of social status specific patterns of oxidative stress in the brain as shown in panel ‘a’. Arrows represent a significant effect (dashed arrow indicates a marginally non-significant effect).

We evaluated the effect of social status on multiple markers of oxidative stress (TAC, total glutathione, oxidative DNA damage) in the telencephalon, midbrain, hindbrain, and hypothalamus (Figure 2a). Here we report *p* values in the text only (for complete statistical results, see Table 1). We found that dominant males had higher levels of TAC than subordinate males in both the midbrain (*p* < 0.002) and hypothalamic region (*p* < 0.005). However, total glutathione levels were significantly higher in the telencephalon in subordinate males compared to dominant males (*p* < 0.02). We further found that dominant males had lower levels of oxidative DNA damage in the midbrain (*p* < 0.006). However, in the hypothalamus the average level of oxidative DNA damage was higher in dominant males than in subordinate males, but we note that this effect was not significant (*p* < 0.07). A schematic summary of patterns of oxidative stress in the brain relative to social status is shown in Figure 2b.

**Table 1:** Statistical results comparing measurements of oxidative stress between dominant and subordinate males.

						Sample sizes	
Marker	Tissue	Estimate	df	*t* value	*P* value	Dominant	Subordinate
TAC	Telencephalon	−0.2185 ± 0.1452	20.00	−1.505	0.148	20	20
	Hypothalamus	−0.145 ± 0.0448	20.00	−3.247	0.00404**	20	20
	Midbrain^1^	−0.376 ± 0.114	40.00	−3.311	0.00198**	20	20
	Hindbrain	0.0655 ± 0.1088	20.00	0.602	0.554	20	20
DNA damage (8-OHdG)	Telencephalon	0.0044 ± 0.007	17.79	0.629	0.537	18	20
	Hypothalamus	−0.013 ± 0.007	20.00	−1.903	0.0715	20	20
	Midbrain	0.0119 ± 0.0038	20.00	3.10	0.00565**	20	20
	Hindbrain	5.135e-04 ± 7.159e-03	38.00	0.072	0.943	29	26
Total GSH	Telencephalon	0.6174 ± 0.2306	16.77	2.677	0.016*	16	17
	Hypothalamus	−0.6655 ± 0.5993	20.00	−1.110	0.28	20	20
	Midbrain	0.1387 ± 0.2145	33.00	0.647	0.522	14	19
	Hindbrain	−0.2343 ± 0.3350	18.97	−0.699	0.493	20	17

1 Effect of body weight was also significant in this model (LMM, body weight: −0.068 ± 0.023, t_40_ = −2.904, *p* = 0.006).

Significant effects are shown in bold. TAC: Total antioxidant capacity; GSH: glutathione.

**Figure 3:**
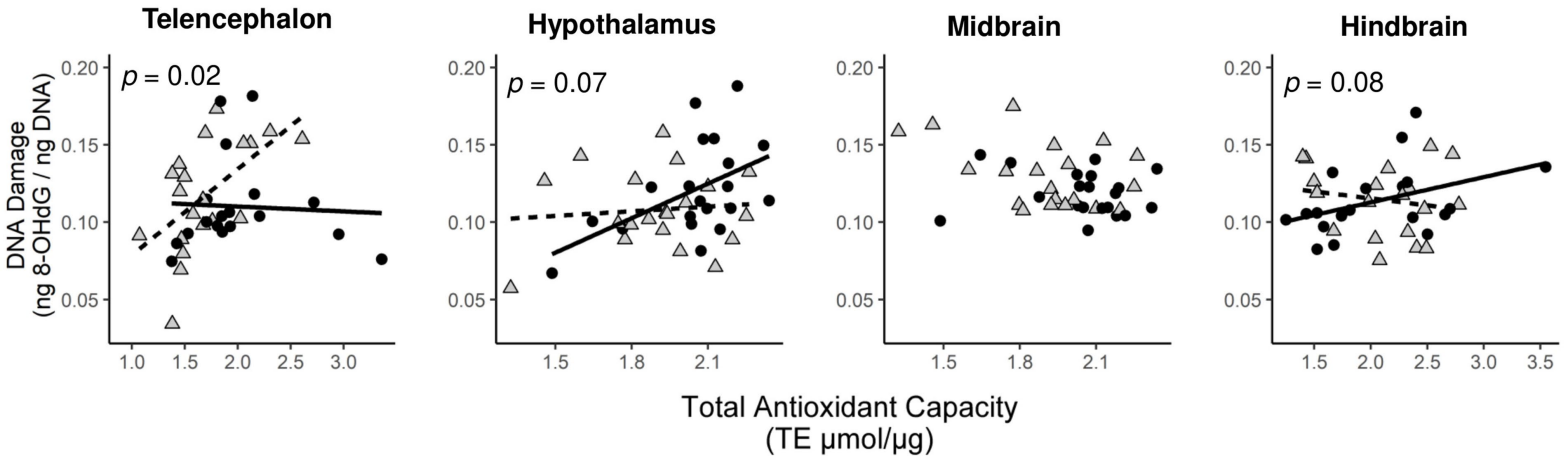
Status-dependent relationship between levels of oxidative DNA damage and total antioxidant capacity in specific brain divisions. Circles are dominant males and triangles are subordinate males. The relationships for dominant males (solid lines) and subordinate males (dashed lines) are shown when the significance level of the interaction effect between total antioxidant function and social status on oxidative DNA damage was *p* < 0.1.

To test for social status specific regulation of oxidative balance, we tested whether variation in oxidative DNA damage was explained by total antioxidant capacity in a social status-specific manner (Figure 3). We found evidence for an interaction effect of social status x TAC on oxidative DNA damage in three brain divisions (Table 2). This effect was significant in the telencephalon (*p* < 0.02) and borderline non-significant in the hypothalamus (*p* < 0.07) and hindbrain (*p* < 0.08). When split by social status, TAC had a significant effect on oxidative DNA damage in several tests (Figure 3; solid lines indicate significant effects), and in all cases this effect was positive.

**Table 2:** Statistical results testing the effect of total antioxidant capacity (TAC) and social status (dominant and subordinate males) on oxidative DNA damage (8-OHdG).

Effect	Brain division	Estimate	df	*t* value	*P* value
Social status x TAC	Telencephalon	0.0455 ± 0.0185	27.25	2.459	0.021*
	Hypothalamus	−0.0616 ± 0.0321	23.06	−1.918	0.0676
	Midbrain	−0.0143 ± 0.0146	28.78	−0.980	0.335
	Hindbrain	−0.0254 ± 0.0143	38.00	−1.777	0.0836

Significant effects are shown in bold.

### Effect of HPG axis activity on oxidative stress

**Figure 4:**
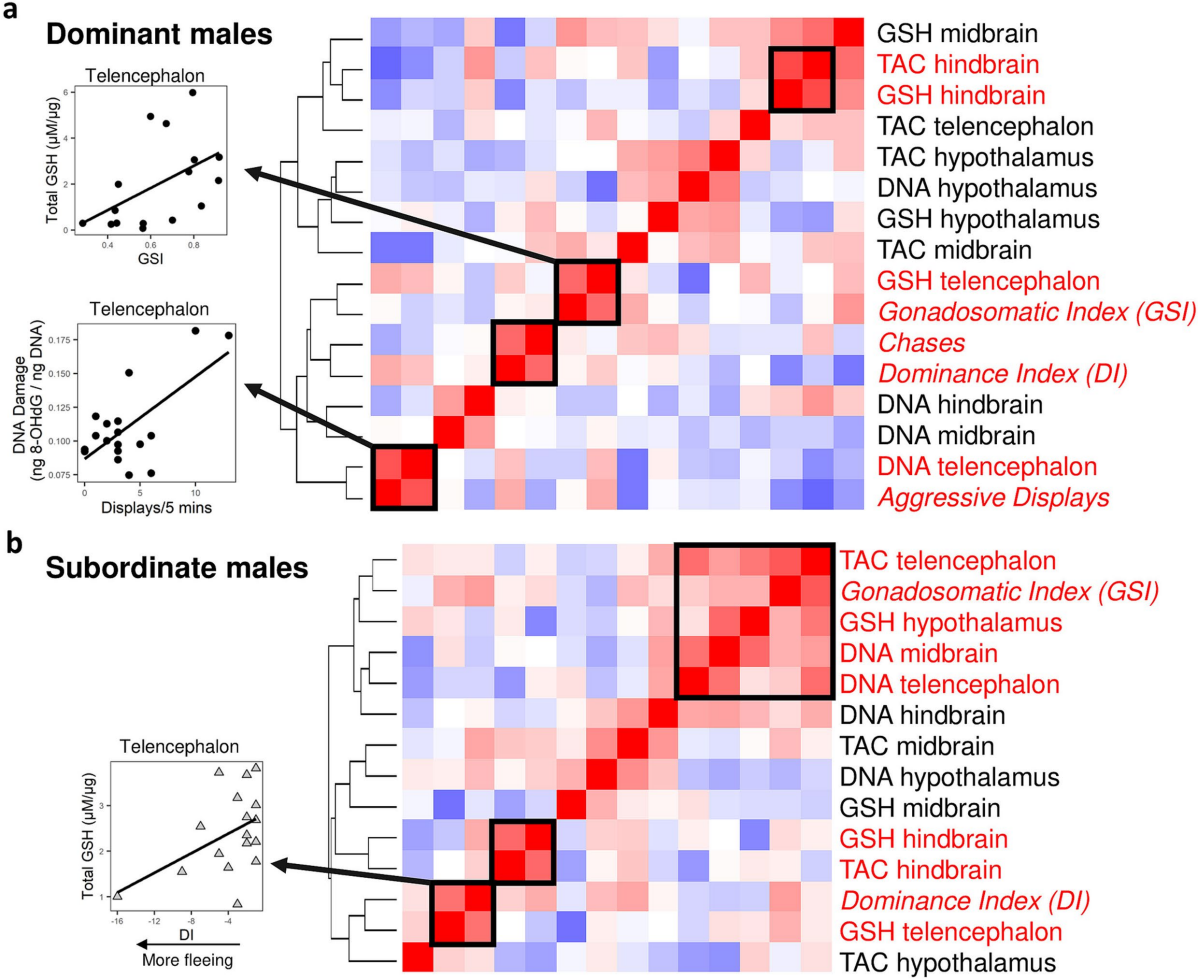
Covariances patterns across markers of oxidative stress combined with indicators of reproduction and social dominance (gonadosomatic index and behavior) for dominant **(a)** and subordinate males **(b)**. For dominant males, we used both the dominance index and measures of overt (chases) and covert aggression (display behaviors). The dominance index is calculated as aggressive behavior + courtship minus flees, and hence in subordinate males lower, more negative dominance index values correspond to more fleeing behavior. For subordinate males, we only used the dominance index due to the lack of aggressive behaviors. Hierarchical clustering revealed significant (indicated by solid box) clusters in both dominant (*n* = 20) and subordinate males (*n* = 20). Each cell represents the correlation value, with red indicating positive correlations and blue indicating negative correlations. Circles are dominant males and triangles are subordinate males. TAC: Total antioxidant capacity; GSH: glutathione; DNA: oxidative DNA damage (8-OHdG).

We performed a hierarchical cluster analysis involving all oxidative stress measurements and two indicators of HPG axis activation: gonadosomatic index (GSI) and dominance index (DI). In dominant males, we also included chases and aggressive display behaviors in the analysis. We found two significant clusters where a marker of oxidative stress co-varied with either GSI, DI, or behavior in both dominant (Figure 4a) and subordinate males (Figure 4b). In dominant males we found that within the telencephalon aggressive display frequency was co-regulated with oxidative DNA damage (hierarchical cluster analysis, *p* = 0.01), and GSI was co-regulated with glutathione levels (*p* = 0.04). Other significant clusters were DI and chase frequency (*p* = 0.02) and TAC and glutathione levels within the hindbrain (*p* = 0.04). In subordinate males, DI (which in subordinate males shows more negative values corresponding to more fleeing behavior) was co-regulated with glutathione levels in the telencephalon (*p* = 0.04). We also found that within subordinate males, GSI was co-regulated with four measurements of oxidative stress in the brain, including TAC and oxidative DNA damage in the telencephalon (*p* < 0.05). Finally, there was a significant cluster containing TAC and glutathione levels within the hindbrain of subordinate males (*p* = 0.01).

## Discussion

We found support for the hypothesis that social status and the extent of HPG axis regulation impacts oxidative stress in the brain. Although circulating oxidative damage is higher in dominant males in our study species (Border et al., 2019), they generally exhibited higher total antioxidant capacity and lower oxidative DNA damage in the brain. However, these patterns were brain division specific, with dominant males showing lower glutathione levels in the telencephalon and a higher levels of oxidative DNA damage in the hypothalamus (this latter effect was marginally non-significant) relative to subordinate males. Social dominance index and gonadosomatic index, which are indicators of HPG axis activity, were co-regulated with some oxidative stress measurements in the brain. Below we will discuss these findings in more detail.

Our findings show that both antioxidant capacity and oxidative DNA damage are impacted by social status but these effects varied by brain division and were marker specific. These general findings are consistent with the idea that distinct social challenges experienced by dominant and subordinate males could impact patterns of oxidative balance in the brain. Social status has been linked to processes in the brain that can alter redox homeostasis, such as differences in synaptic plasticity (Behrens et al., 2007; Hasam-Henderson et al., 2018) as well as rank-specific activation of neural circuits that regulate social behavior (Fernald and Maruska, 2012; Weitekamp et al., 2017). Here we focus on the possibility that social status effects on oxidative stress in the brain are mediated by social status-specific activation of neuroendocrine systems, such as the HPA axis and the HPG axis.

The reduced antioxidant capacity in subordinate males in two brain divisions is consistent with the idea that social stress may impair redox balance in the brain. Chronic psychosocial stress experienced by socially subordinate individuals increases basal levels of glucocorticoids (Creel, 2001; Abbott et al., 2003; Goymann and Wingfield, 2004; Sapolsky, 2004; Creel et al., 2013; Larrieu and Sandi, 2018), which is also the case in *A. burtoni* with subordinate males typically exhibiting higher levels of cortisol than dominant males (Fox et al., 1997). High levels of glucocorticoids make neurons across the brain more susceptible to oxidative insults via well-described actions of glucocorticoids, including downregulating several antioxidant enzymes and increasing baseline levels of ROS production (Şahin and Gümüşlü, 2004; Spiers et al., 2015; Torres et al., 2021). The increased oxidative DNA damage in the midbrain in subordinate males compared to dominant males is consistent with social subordination impairing the ability to maintain proper redox homeostasis in specific parts of the brain.

However, there was also some support for an oxidative cost of social dominance: dominant males tended to have higher levels of oxidative DNA damage (but note that this effect was non-significant) in the hypothalamus and had reduced glutathione levels in the telencephalon. As stated earlier, dominant *A. burtoni* males have an upregulated reproductive HPG axis and increased androgen levels to facilitate reproduction (mate searching, courtship, gonadal growth). Androgens can directly modulate oxidative balance since androgens can have both neuroprotective and neurotoxic effects (Bueno et al., 2017; Snyder et al., 2018; Chunchai et al., 2019) depending on the oxidative state of specific neurons (Duong et al., 2020). It is possible that social status differences in circulating androgens at least in part mediate differences in oxidative stress between dominant and subordinate males.

To further examine how activation of the HPG axis regulates patterns of oxidative stress in the brain, we took advantage of the fact that both dominant and subordinate males exhibit individual variation in indicators of HPG axis activity, the dominance index (DI, essentially a measure of the strength of territoriality), aggressive behavior (chases and aggressive displays) and gonadosomatic index (GSI). Both DI and GSI were co-regulated with markers of oxidative stress in specific brain divisions in both dominant and subordinate males. In dominant males, GSI was co-regulated with glutathione levels in the telencephalon and aggressive displays were co-regulated with oxidative damage in the telencephalon. In subordinate males, DI was co-regulated with glutathione levels in the telencephalon. The fact that these associations occurred in the telencephalon supports our suggestion that HPG axis regulation and androgens modulate oxidative balance, because the telencephalon of *A. burtoni* expresses a high density of androgen and estrogen receptors (Burmeister et al., 2007), and hence it would be easy to expect a potentially stronger androgenic regulation of oxidative stress in this part of the brain (possibly via estrogenic signaling after the local conversion of androgens into estrogens). It should be noted however, that glucocorticoid signaling may also explain some of these associations. For example, in subordinate males we found that more negative DI scores, which reflect more fleeing behavior, were linked to reduced glutathione levels in the telencephalon. This pattern is consistent with the high density of glucocorticoid receptors in the teleost telencephalon (Natsaridis et al., 2023) and the well-described negative effects of glucocorticoids on brain antioxidant function (McIntosh et al., 1998). Our findings are consistent with a previous study where the rate of social dominance behavior, which is influenced by the HPG axis, was positively related to total antioxidant function in the brain of the cichlid fish *Neolamprologus pulcher* (Culbert et al., 2022). An effect of HPG axis regulation on oxidative balance in the brain is also supported by brain transcriptomics data showing the pathways involved in metabolism and redox homeostasis are coregulated with indicators of HPG axis activation in *A. burtoni* (Peter Dijkstra and Ryan Wong, unpublished data).

To specifically test whether HPG axis activation influences oxidative stress in the brain, future studies should directly manipulate HPG axis using pharmacology (O’Connell and Hofmann, 2012) or genetically altered *A. burtoni* where specific androgen receptors have been disrupted (Alward et al., 2020). We note that in the current study, circulating androgen levels were not measured. Although gonadosomatic index and dominance index are correlated with androgen levels (Dijkstra, unpublished data), directly linking androgen levels to patterns of oxidative stress in the brain will be an important future direction. Furthermore, while our approach provides important insight into broad-scale patterns of brain oxidative stress, we may have missed regional differences in oxidative stress. Future studies should therefore define the effect of social status and HPG axis regulation on oxidative damage in specific brain regions that regulate adaptive responses to social challenges. Ideally, these brain region specific patterns of oxidative stress should be linked to variation in androgen or estrogen receptor density in specific cells of the nervous system (e.g., neurons vs. glial cells) as well as circulating levels of androgens.

There are additional mechanisms that could induce higher levels of oxidative stress in specific parts of the brain of dominant males. Dominant males face greater cognitive demands than subordinate males because they need to be more attentive and responsive to changes in the social landscape while maintaining their position in the hierarchy (Sapolsky, 2005). Accordingly, adult neurogenesis is typically higher in dominant males than subordinate males across vertebrates (Maruska et al., 2012; Holmes, 2016; Tea et al., 2019), including *A. burtoni*. Since adult neurogenesis can increase ROS generation (Walton et al., 2012), dominant males may be susceptible to localized oxidative stress in regions of the brain where neurogenesis occurs (e.g., the hippocampus).

Although our study was largely framed within the context of social stress and redox balance in the brain, it would be interesting to investigate how patterns of oxidative stress in the brain are relevant to Darwinian fitness and behavioral decision making in a natural context. The cichlid fish *A. burtoni* inhabits Lake Tanganyika and associated river systems (Fernald and Hirata, 1977). It lives in shallow shore pools and rivers where males compete aggressively for social dominance and territory ownership which is linked to mating success. The patterns of oxidative stress in the brain could influence the relative cost and benefits of high and low social status in cichlid dominance hierarchies. Both dominant (territorial) and subordinate (non-territorial) status have a distinct cost (Hofmann et al., 1999; Dijkstra et al., 2013). For example, in *A. burtoni* dominant males grow slower than subordinate males (Hofmann et al., 1999), dominant males were found to display higher levels of circulating oxidative damage than subordinate males (Border et al., 2019; Fialkowski et al., 2021), and the level of territorial defense was linked to circulating oxidative damage in a color phenotype dependent manner (Border et al., 2019; Dijkstra et al., 2024). Our study suggests that we may need to consider oxidative stress in the brain when investigating the costs of high and low rank in group-living organisms. We also note that the oxidative cost of social dominance and reproduction has received considerable interest in the field of evolution and life history theory aimed at understanding the trade-off between competing life history traits, such as reproduction and lifespan (Beaulieu et al., 2014; Sharick et al., 2015; Marasco et al., 2017). Given the importance of the brain for making optimal behavioral decisions and the impact of redox balance in the brain on behavior (Vicente-Gutierrez et al., 2019), improving our understanding of how demanding activities impact oxidative balance in the brain is a fruitful avenue for future studies to those who are interested in the social determinants of Darwinian fitness and health.

We have previously shown that social status and reproduction affect patterns of oxidative stress in the peripheral tissue of cichlid fish (Border et al., 2019; Sawecki et al., 2019; Fialkowski et al., 2021; Culbert et al., 2023). In the current study, we expand on these findings to show that social status also influences patterns of oxidative stress in the central nervous system. Cumulative oxidative damage is one of the main mechanisms determining vulnerability to depression and driving age-related changes in neurodegenerative disease (Kandlur et al., 2020). A deeper understanding of the sources of oxidative stress during social challenges, and how innate defensive mechanisms are activated across the entire brain is critical for expanding our understanding of how brain health is maintained in the face of social stress and aging (Liu et al., 2017). Our study lays a foundation for future research on the effect of social competition on oxidative stress in the brain in a tractable model organism, amenable to social manipulation (Rodriguez-Santiago et al., 2020; Piefke et al., 2021).

## Data Availability

The raw data supporting the conclusions of this article will be made available by the authors upon request.
